# Predicting spatial variability of species diversity with the minimum data set of soil properties in an arid desert riparian forest

**DOI:** 10.3389/fpls.2022.1014643

**Published:** 2022-11-11

**Authors:** Xiaotong Li, Yudong Chen, Guanghui Lv, Jinlong Wang, Lamei Jiang, Hengfang Wang, Xiaodong Yang

**Affiliations:** ^1^ College of Ecology and Environment, Xinjiang University, Xinjiang, China; ^2^ Key Laboratory of Oasis Ecology of Education Ministry, Xinjiang University, Xinjiang, China; ^3^ Xinjiang Jinghe Observation and Research Station of Temperate Desert Ecosystem, Ministry of Education, Jinghe, China; ^4^ School of Civil & Environmental Engineering and Geography Science, Ningbo University, Ningbo, China

**Keywords:** Geo-statistics, Random Forest model, spatial distribution, spatially heterogeneous, desert riparian forests

## Abstract

Species diversity has spatial heterogeneity in ecological systems. Although a large number of studies have demonstrated the influence of soil properties on species diversity, most of them have not considered their spatial variabilities. To remedy the knowledge gap, a 1 ha (100 m × 100 m) plots of arid desert riparian forest was set up in the Ebinur Wetland Nature Reserve (ELWNR) in the NW China. Then, the minimum data set of soil properties (soil MDS) was established using the Principal Component Analysis (PCA) and the Norm Value Determination to represent the total soil property data set (soil TDS). The Geo-statistics and two models (*i.e.*, Random Forest/RF and Multiple Linear Regression/MLR) were used to measure the spatial variability of species diversity, and predict its spatial distribution by the soil MDS, respectively. The results showed that the soil MDS was composed of soil salt content (SSC), soil total phosphorus (STP), soil available phosphorus (SAP), soil organic carbon (SOC) and soil nitrate nitrogen (SNN); which represented the soil TDS perfectly (*R^2 =^* 0.62). Three species diversity indices (*i.e.*, Shannon–Wiener, Simpson and Pielou indices) had a high spatial dependence (C_0_/(C_0_+C)< 25%; 0.72 m ≤ range≤ 0.77 m). Ordinary kriging distribution maps showed that the spatial distribution pattern of species diversity predicted by RF model was closer to its actual distribution compared with MLR model. RF model results suggested that the soil MDS had significant effect on spatial distribution of Shannon–Wiener, Simpson and Pielou indices (*Var_ex_* = 56%, 49% and 36%, respectively). Among all constituents, SSC had the largest contribution on the spatial variability of species diversity (nearly 10%), while STP had least effect (< 5.3%). We concluded that the soil MDS affected spatial variability of species diversity in arid desert riparian forests. Using RF model can predict spatial variability of species diversity through soil properties. Our work provided a new case and insight for studying the spatial relationship between soil properties and plant species diversity.

## 1 Introduction

Species diversity inseparably links to ecosystem stability (Ruiz-Jaén and Aide, 2005; Wortley et al., 2013; Hamberg et al., 2020). Studies of species diversity contribute to a full understanding of the ecosystem balance of structures and functions (Cardinale et al., 2002; Chen et al., 2006), as well as the dynamic change of the community (Kang et al., 2010). Currently, a great number of studies have focused on the hot spots with rich biodiversity, while remote and species**-**poor areas, like arid desert, have lagged behind hot spots (Ma et al., 1995; He et al., 1998; Ricotta and Avena, 2003; Li et al., 2018).

As one of the most fragile biotic types, desert ecosystems are gifted with distinctive regional flora and a large number of characteristic species. Despite they possess relatively low productivity and species diversity (Waide et al., 1999), desert ecosystems are home to rare and relict plant species that can tolerate extreme environment (*i.e.*, scarce rainfall, high salt content and poor-soil nutrients) (Ezcurra, 2006). Understanding the changes and the influencing mechanisms of species diversity in deserts will benefit plant conservation at the global level. At present, many studies have showed that species diversity is mainly determined by soil factors in the arid desert ecosystem (Wang et al., 2009; Midgley, 2012; Jin et al., 2021a). However, soil factors are numerous and interrelated, making it difficult to figure out the relationship between species diversity and soil properties (Ma et al., 2021). Researchers want to select a small number of indicators to provide an integrated, comprehensive, and accurate view of the overall soil properties, while maximizing the inclusion of all relevant information about the soil properties and minimizing data redundancy (Andrews et al., 2004).

Larson and Pierce (1991) proposed the concept of a minimum data set of soil properties (soil MDS), whose core function is to establish a set of simplified and practical data (including few properties) to replace a large number of complex soil data, and to better grasp the whole characteristics or status of soils (Yu et al., 2018; Li et al., 2019; Tian et al., 2020). Such method saved the substantial cost of time and labor for measurement of all soil properties, and reduce data redundancy due to collinearity among different properties (Li et al., 2019). The researches in soil quality assessment and plant-soil relationship demonstrated the results based on the soil MDS were superior to that based on the total soil property data set (soil TDS) (Volchko, 2014; Rahmanipour et al., 2014; Wu et al., 2019). Therefore, using the soil MDS as key proxy of all soil properties, may make it more convenient and efficient to study the relationship between soil properties and species diversity (Rezaei, 2006; Wu et al., 2019; Jin et al., 2021b). However, our understanding of the relationship between species diversity and the soil MDS in arid deserts is still unclear.

Many studies have shown that soil properties (*e.g.* soil nitrogen content, soil water content, phosphorus content and pH) have significant effects on plant species diversity (Ceulemans et al., 2014; Wang et al., 2015; Palpurina et al., 2017). It is well known that the spatial distribution of soil properties and their effects on ecological processes, such as biodiversity maintenance, are often spatially heterogeneous (Dutilleul and Legendre, 1993; Fortin and Payette, 2002; Wang et al., 2021). Geo-statistics has been widely developed in ecological research in recent years (Robertson, 1987; Kent et al., 2006). It not only reveals the spatial patterns, variability and relevant properties of attribute variables, but also links spatial variability with ecological processes, as well as clarifies their influencing factors (Wang et al., 2021). However, the vast majority of current studies about species diversity have not addressed spatially heterogeneous, especially for arid desert ecosystems (Lu et al., 2018). Furthermore, we do not know the underlying causes of spatial heterogeneity in species diversity and whether it is influenced by the soil MDS.

Desert riparian forests are an important and special community type in continental arid ecosystems, which are unique bio-systems with the most active life phenomenon, high biodiversity and primary productivity (Damasceno-Junior et al., 2005; Kang et al., 2021). Since the number of species in riparian forests is much higher than that in typical xerophytic sparse shrubs, it is considered as a key biodiversity conservation area in arid desert (Chen et al., 2022). On the other hand, affected by global warming and human activity, such as groundwater extraction and deforestation, the area of riparian forests has been shrinking rapidly over the past few decades (Zeng et al., 2020). Revealing the spatial distributions and influencing mechanisms of species diversity in riparian forests has become one of the key points of species conservation and forest restoration in arid desert areas (Kang et al., 2021). The Ebinur Lake Wetland Nature Reserve (ELWNR) is located in the northwestern China. Affected by harsh environments (*i.e.*, low soil water and nutrient availability, high soil salinity and frequent wind disturbance), ecosystem in the ELWNR is highly sensitive and fragile to environmental changes (Wang et al., 2021). In contrast, the ELWNR is a resource treasury of biodiversity in arid desert areas (Jiang et al., 2022), which has well-protected and typical riparian forests in northwest China. It offers an eligible place for investigating the spatial relationship between species diversity and soil properties. The objectives of our work are to reveal the spatial variability of species diversity in arid desert riparian forest and to evaluate the complicated spatial relationships between species diversity and the soil MDS. The detailed objectives of the study are as follows: (1) to find a soil MDS for the study area; (2) to identify the relationship between the soil MDS and species diversity; (3) to predict the spatial distribution of species diversity from the soil MDS.

## 2 Materials and methods

### 2.1 Study area

The study was conducted in the ELWNR (44°30’–45°09’N, 82°36’–83°50’E), which is located in the southern part of the Gurbantünggüt Desert in the Xinjiang Uygur Autonomous Region, NW China. The study area features a typical northern temperate continental arid climate, with the mean annual precipitation and evaporation less than 100 mm and more than 1500 mm, respectively (Yang et al., 2019). The mean annual temperature is 7.8°C. The maximum and minimum temperatures in summer and winter can achieve 36.4°C and -41.3°C in extreme situation, respectively (Wang et al., 2021). Due to the scarce precipitation, soil water is primarily recharged by rivers and groundwater (Li et al., 2006). Affected by the low local vegetation cover and forest biomass, the organic matter content in the soil is relatively low, with values ranging from 0.28% to 5.46% (Qin et al., 2011).

The typical soil types in the study area are mainly gray desert soil, aeolian sand soil and gray brown desert soil (Wang et al., 2013; Token et al., 2022). The varied soil types have supported abundant communities of plant, in particular of xerophytic desert species. *Phragmites australis* Trin. ex Steud., *Apocynum venetum* L., *Halimodendron halodendron* Dum. Cours., *Nitraria tangutorum* Bobr., *Achnatherum splendens* Nevski, *Lycium ruthenicum* Murray, *Populus euphratica* Oliv., *Suaeda microphylla* Pall., *Alhagi camelorum* Fisch., *Reaumuria soongonica* Pall., *Haloxylon ammodendron* Bunge, *Salsola collina* Pall., *Sonchus oleraceus* L. and *Glycyrrhiza uralensis* Fisch. are the main species in the study area. Frequencies of plant species are presented in the **Supplementary Table S1**.

### 2.2 Soil sampling and measurements

#### 2.2.1 Sampling site

A total of 7 rivers with annual runoff over 1× 10^8^ m^3^·a^-1^ are included in the ELWNR, which makes the local desert riparian forest distribute in a very wide range, accounting for about 58.44% of the total area of the reserve. Among all rivers, the Aqikesu River flows through the largest area, making its banks form the largest and most typical riparian forest in the ELWNR. In this study, in order to survey more plants, the peak season of local plant growth (from July to August) in 2018 was selected to set up sampling plot and survey community.

**Figure 1:**
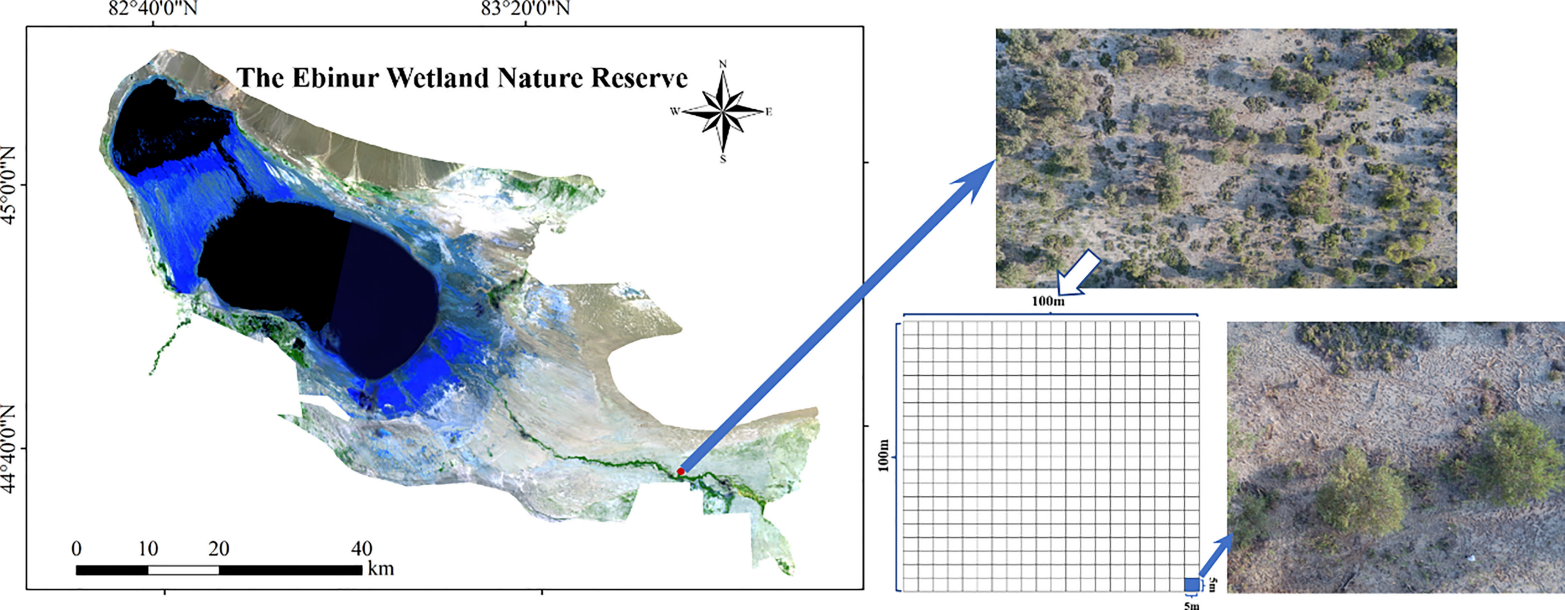
Sampling sites and the division of 1 ha sampling plot.

A 1 ha sampling plot (100 m × 100 m) of plant community was conducted in riparian forest on the north bank of the Aqikesu River. Then, the plot was divided into 400 continuous quadrats with the area of 5 m × 5 m (25 m^2^) (**Figure 1**). Vegetation surveys were carried out within each quadrat. Species names, plant abundances, geographical coordinates and elevation of each quadrat were recorded. Here 1 ha plot was selected for the study of spatial heterogeneities of species diversity and the soil MDS, because this area covered most of the plant species and community types in the desert ecosystem (Yuan et al., 2018). At the same time, the boundary length of 1 ha plots (100 m) was larger than the spatial variation distance (or range) of soil properties (Lu et al., 2018; Wang et al., 2021). The division of the 1 ha plot into 400 small quadrats with an area of 25 m^2^ was based on the need for spatial geographic statistical analysis. In the analysis of spatial heterogeneity of species diversity, it is required that the analytic objects (geographic grids) are adjacent to each other, and located in different spatial positions. Each grid also has its own unique attributes (which refers to the species diversity index in this study) (Wang and Yang, 2022). Unlike the 100 m^2^ or 400 m^2^ sampling area of many temperate forests or mountain coniferous forests, the grid or small quadrat area was defined as 25 m^2^ because it was found to be the minimum sampling area for the desert riparian forests in arid areas (Wang and Guo, 2016; Kong et al., 2016).

#### 2.2.2 Sampling and measurements

Sampling points were established at the center of the quadrats for soil samples collections. As suggested by Tian et al. (2010), 0-20 cm topsoil was set as our sampling object because it enriched the vast majority of soil nutrients. The samples were transferred into the corresponding aluminium boxes, weighed and taken back to the laboratory. The soil samples were then dried and weighed to calculate soil water content (SWC). Meanwhile, another soil samples were also collected, and then passed through a 2-mm sieve in the laboratory for the further measurements of soil properties (**Table 1**) (Bao, 2000).

**Table 1:** Measurement methods of soil properties.

Soil properties	Abbr.	Measuring method
Soil water content	SWC (g·kg^−1^)	Drying method
Soil salt content	SSC (g·kg^−1^)	Electrical conductivity method
pH	pH	Glass electrode method
Soil organic carbon	SOC (g·kg^−1^)	Potassium dichromate method
Soil total nitrogen	STN (g·kg^−1^)	Kjeldahl nitrogen method
Soil ammonium nitrogen	SAN (mg·kg^−1^)	Indophenol blue colorimetry
Soil nitrate nitrogen	SNN (mg·kg^−1^)	Dual-wavelength ultraviolet spectrophotometry
Soil total phosphorus	STP (g·kg^−1^)	Molybdenum blue colorimetric method
Soil available phosphorus	SAP (mg·kg^−1^)	Molybdenum antimony anti-colorimetric method

### 2.3 Data processing and statistical analysis

#### 2.3.1 Calculation of species diversity indices

The Shannon–Wiener diversity index, Simpson diversity index and Pielou evenness index were selected as proxies for species diversity due to their universality and high popularity (Simpson, 1949; Spellerberg and Fedor, 2003). The calculations are as following:

Shannon–Wiener diversity index:


Equation 1 $$H^{\prime }=-\sum_{i=1}^{S}\left(P_{\text{i}}\ln P_{i}\right)\;$$

Simpson diversity index:


Equation 2 $$\begin{array}{c} D=1-\sum_{i=1}^{S}{\left(P_{i}\right)}^{2} \end{array}$$

Pielou evenness index:


Equation 3 $$\begin{array}{c} \;\;\;\;\;\;\;\;J=H^{\prime }/\ln S\; \end{array}$$

where *S* is the total number of species in a unit, *P*_*i*_ is the proportion of the abundance of the *i* th species relative to the total abundance.

#### 2.3.2 The soil MDS selection and verification

The combination of Principal Component Analysis (PCA) with Norm Value Determination was used to obtain the pivotal constitutors (or indicators) of the soil MDS from the soil TDS (Yu et al., 2018; Tian et al., 2020). The grouping process of the PCA was referred to Jin et al. (2021b). Norm Values are the comprehensive loadings of given indicators across all components, which were calculated to screen the indicators. Norm values were calculated separately for each soil property according to Equation 4. Indicators with norm values within the top 10% of the maximum norm value in each group were temporarily retained. The Pearson correlation analysis was then used to select indicators. If these indicators were significantly correlated, then the one with the highest norm value was retained in the soil MDS, and all others were removed. Conversely, the uncorrelated indicators were retained in the soil MDS (Wu et al., 2019; Jin et al., 2021b).


Equation 4 $$\begin{array}{c} N_{ik}=\sqrt{\sum_{i=1}^{k}\left(U_{ik}^{2}\lambda_{k}\right)\;} \end{array}$$

where *N*_*ik*_ is the comprehensive loading of indicator *i* in all components with eigenvalues ≥ 1, *U*_*ik*_ is the loading of indicator *i* in component *k* , and is the eigenvalue of component *k*.

The soil property index (*SPI*) was used to verify the similarity between the soil MDS and the soil TDS, or to test the feasibility of using the soil MDS instead of the soil TDS. The specific method was to analyze the relationship between soil MDS and TDS by *SPI* using Linear Regression. *SPI* was calculated from the weights of the soil property indicators (*W_i_*) and the score of indicators *i* [*F(X_i_*)] in Membership Functions (Equation 5-7) (Qi et al., 2009). More specific, *W_i_* was the ratios of the common factor variances to the sum of the common factor variances (Shukla et al., 2006; Jin et al., 2021b), which obtained from PCA (Zhang et al., 2022).


Equation 5 $$\begin{array}{c} \;SPI=\sum_{i}^{n}W_{i}\times F(X_{i}) \end{array}$$

*F(X_i_*)s in the Membership functions were created on the basis of the plus and minus effects of the indicators. The soil indicators were transformed into dimensionless scores ranging from 0.00 to 1.00 (Jin et al., 2021b). Then, *F(X_i_*)s were calculated by Equation 5 and 6 according to the different data types, respectively:


Equation 6 $$\begin{array}{c} F(X_{i})=\{\begin{array}{c} 0\;\;\;\;\;\;\;\;\;\;\;\;\;\;\;\;\;\;\;\;\;\;\;\;X_{i}\leq X_{Min}\\ \frac{X_{i}-X_{Min}}{X_{Max}-X_{Min}}\;\;\;\;\;\;\;\;X_{Min}<X_{i}<X_{Max}\\ 1\;\;\;\;\;\;\;\;\;\;\;\;\;\;\;\;\;\;\;\;\;\;\;\;X_{i}\geq X_{Max}\; \end{array} \end{array}$$

Equation 7 $$F\left(X_{i}\right)=\{\begin{array}{c} 0\;\;\;\;\;\;\;\;\;\;\;\;\;\;\;\;\;\;\;\;\;\;\;\;X_{i}\geq X_{Max}\\ \frac{\;(X_{Max}-X_{i})}{X_{Max}-X_{Min}}\;\;\;\;\;\;\;\;X_{Min}<X_{i}<X_{Max}\\ 1\;\;\;\;\;\;\;\;\;\;\;\;\;\;\;\;\;\;\;\;\;\;\;\;X_{i}\leq X_{Max}\; \end{array}$$

where *X*_*i*_ is the actual value of the indicators; *X_Min_* and *X_Max_* are the lower and upper limit of the critical value of the indicator, which represent the minimum and maximum values measured in the actual environment, respectively (Wu et al., 2019; Jin et al., 2021b).

#### 2.3.3 Geo-statistics

The semi-variograms of geo-statistics were applied to obtain the structural characteristics of spatial variation of species diversity in the ELWNR, which was calculated as Equation 8 (Robertson et al., 1993):


Equation 8 $$\begin{array}{c} \gamma (h)=\frac{1}{2N(h)}\sum_{i=1}^{N(h)}{\left[Z(x_{i})-Z(x_{i}+h)\right]}^{2}\; \end{array}$$

where *N(h)* is total number of sample couples for the separation distance *h*, and *Z*(*x*_*i*_) and *Z*(*x*_*i*_+*h*) are measured sample values at points *i* and *i+h*, respectively.

The nugget to sill ratio [C_0_/(C_0_+C)] is commonly described as variable spatial dependence (Vasu et al., 2017). As Cambardella et al. (1994) stated, the ratios below 25%, between 25% and 75% and over 75% signify strong, moderate and weak spatial dependence, respectively. Range value was the largest distance of autocorrelation or spatial dependence (Behera et al., 2018).

Four commonly theoretical models, *i.e.*, exponential, Gaussian, linear and spherical models, were used to fit semi-variograms. High coefficient of determination (*R* ^2^) indicated a good fitting of model. In addition, ordinary kriging interpolation plots were used to select the best-fitting model *via* the visual inspection (Hou et al., 2021). Here the Ordinary kriging was adopted in spatial interpolation because it provided the unbiased predictions for specific un-sampled sites and minimized the effect of outliers (Bogunovic et al., 2017; Vasu et al., 2017; Fu et al., 2018; Gao et al., 2019; Hou et al., 2021). All maps were produced with GS + (Version 9.0).

#### 2.3.4 Model prediction

The relationship between species diversity and the soil MDS was fitted using Multiple Linear Regression (MLR) and Random Forest (RF) models. Details on the MLR and RF model can be found in Yilmazer and Kocaman (2020), and Chagas et al. (2016), respectively. The operation process of these two models were accomplished respectively in the ‘*stats*’ and ‘*rfPermute*’ packages of R software (R Core Team, 2019).

Two models’ performance were evaluated and compared using root mean square error (RMSE) and mean relative error (MRE) (Equations 9 and 10). The optimal model was selected in consideration of the lowest RMSE and MRE. Where $T_{i}^{'}$ and *T*_*i*_ are the predicted and observed species diversity indices, respectively, and *n* represents the number of measurements.


Equation 9 $$\begin{array}{c} RMSE=\sqrt{\frac{1}{n}\sum_{i=1}^{n}{\left(T_{i}^{'}-T_{i}\right)}^{2}} \end{array}$$

Equation 10 $$\begin{array}{c} MRE=\frac{1}{n}\sum_{i=1}^{n}\frac{\left|T_{i}^{'}-T_{i}\right|}{T_{i}} \end{array}$$

## 3 Results

### 3.1 Statistical analyses and the soil MDS establishment

The descriptive statistics of soil properties were presented in **Table 2**. CV is the ratio of standard deviation to mean value, which represents the standardized variability. CV values for soil properties ranged from 3.90% (pH) to 57.20% (SOC). According to Hillel (1980) and Nielsen and Bouma (1985), soil properties can be classified into two types based on the CV values: the low (CV< 10% for pH); and the moderate variabilities (CV from 10 to 100% for SWC, STP, SSC, SOC, SAP, STN, SAN and SNN).

**Table 2:** Descriptive statistics of soil properties.

Indicators	Maximum	Minimum	Mean ± SD	SE	CV (%)	Normal distribution
SWC (g·kg−1)	26.11	5.18	13.12 ± 3.70	0.19	18.50	L.N.
SSC (g·kg^−1^)	10.21	1.33	5.59 ± 2.44	0.12	43.60	N
pH	8.94	7.22	8.07 ± 0.32	0.02	3.90	L.N.
SOC (g·kg^−1^)	27.73	2.04	9.57 ± 5.51	0.27	57.20	N
SAP (mg·kg^−1^)	89.76	11.67	38.19 ± 14.99	0.75	39.10	L.N.
STP (g·kg^−1^)	2.17	0.83	1.31 ± 0.25	0.01	19.30	N
STN (g·kg^−1^)	9.98	0.51	2.05 ± 0.75	0.04	36.60	L.N.
SAN (mg·kg^−1^)	10.21	0.60	2.48 ± 1.27	0.06	51.60	L.N.
SNN (mg·kg^−1^)	43.97	2.03	12.51 ± 7.12	0.36	57.00	L.N.

SD, Standard Deviation; SE, Standard Error; CV, Coefficient of variation; N., normal distribution; L.N., normal distribution using logarithmic transformation.

Results of *K-S* test presented that the SSC, SOC and STP followed normal distribution, suggested that these soil properties have uniform variances and no outliers. The data of SWC, pH, SAP, STN and SAN were transformed using logarithmic transformation due to the fact that they had not followed the normal distribution (**Table 2**).

**Figure 2:**
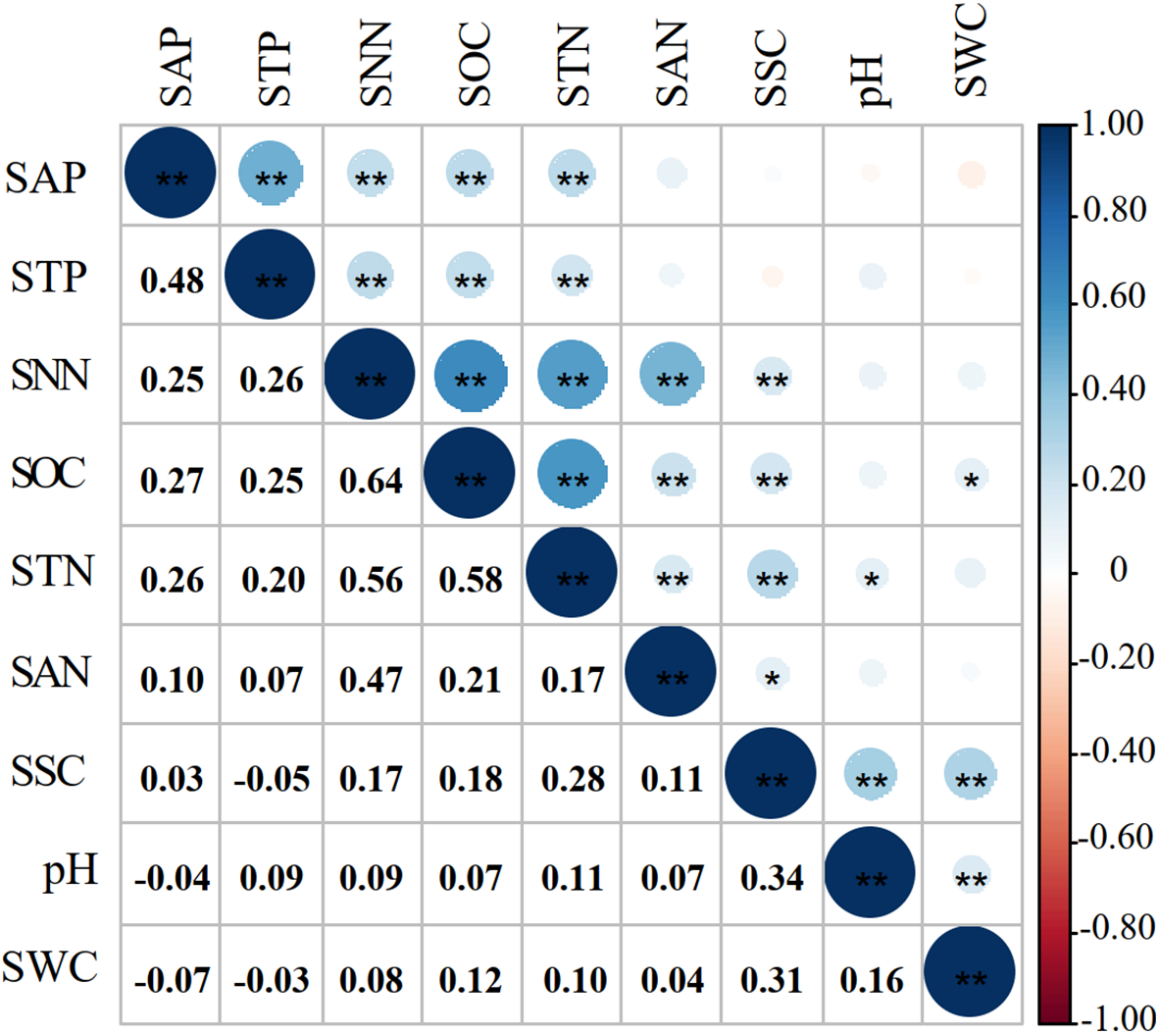
Correlation coefficients of soil properties. * and ** indicate a significant correlation at the 0.05 and 0.01, respectively.

**Figure 2** showed that most of the correlation coefficients between the soil properties were significant at the 0.01 and 0.05 levels, indicating that the soil TDS existed redundant (Wu et al., 2019). In this case, the soil MDS can be obtained from the soil TDS.

Three extracted principal components (PC) with eigenvalues ≥ 1 explained 60.75% of the cumulative contribution (**Table 3**). The load value of SSC in PC2 was greater than 0.5, while the load value of STP in PC3 was greater than 0.5. The retained soil properties were assigned to the third group (PC3). According to the principle that the top 10% of the maximum in each group entered the soil MDS, SSC and STP in the first and second groups entered the soil MDS. In the third group, SNN (0.89) with the largest norm value entered the MDS. SAP (0.82) and SOC (0.81), with norm values in the top 10% of the maximum norm value (SNN, 0.89), entered the soil MDS. Correlation coefficients between SAP, SOC, and SNN were less than 0.5 (**Figure 2**). A total of five soil properties (*i.e.*, SSC, STP, SAP, SOC and SNN) were entered in the soil MDS.

**Table 3:** Load matrix and norm values of indicators.

	Principal component variable			Group	Norm value	Minimum data set
	PC1	PC2	PC3			
SWC	0.12	0.48	0.17	3	0.65	/
SSC	0.23	0.53	0.21	1	0.79	Enter
pH	0.14	0.42	0.40	3	0.72	/
SOC	0.48	−0.03	−0.14	3	0.81	Enter
SAP	0.29	−0.41	0.41	3	0.82	Enter
STP	0.27	−0.38	0.51	2	0.85	Enter
STN	0.46	0.04	−0.06	3	0.77	/
SAN	0.28	0.03	−0.48	3	0.68	/
SNN	0.50	−0.04	−0.31	3	0.89	Enter
Eigenvalues	2.79	1.56	1.12	/	/	/
Variance contribution (%)	31.03	17.33	12.39	/	/	/
Cumulative variance contribution (%)	31.03	48.36	60.75	/	/	/

“/” means failure to enter the soil MDS or no related data.

**Figure 3:**
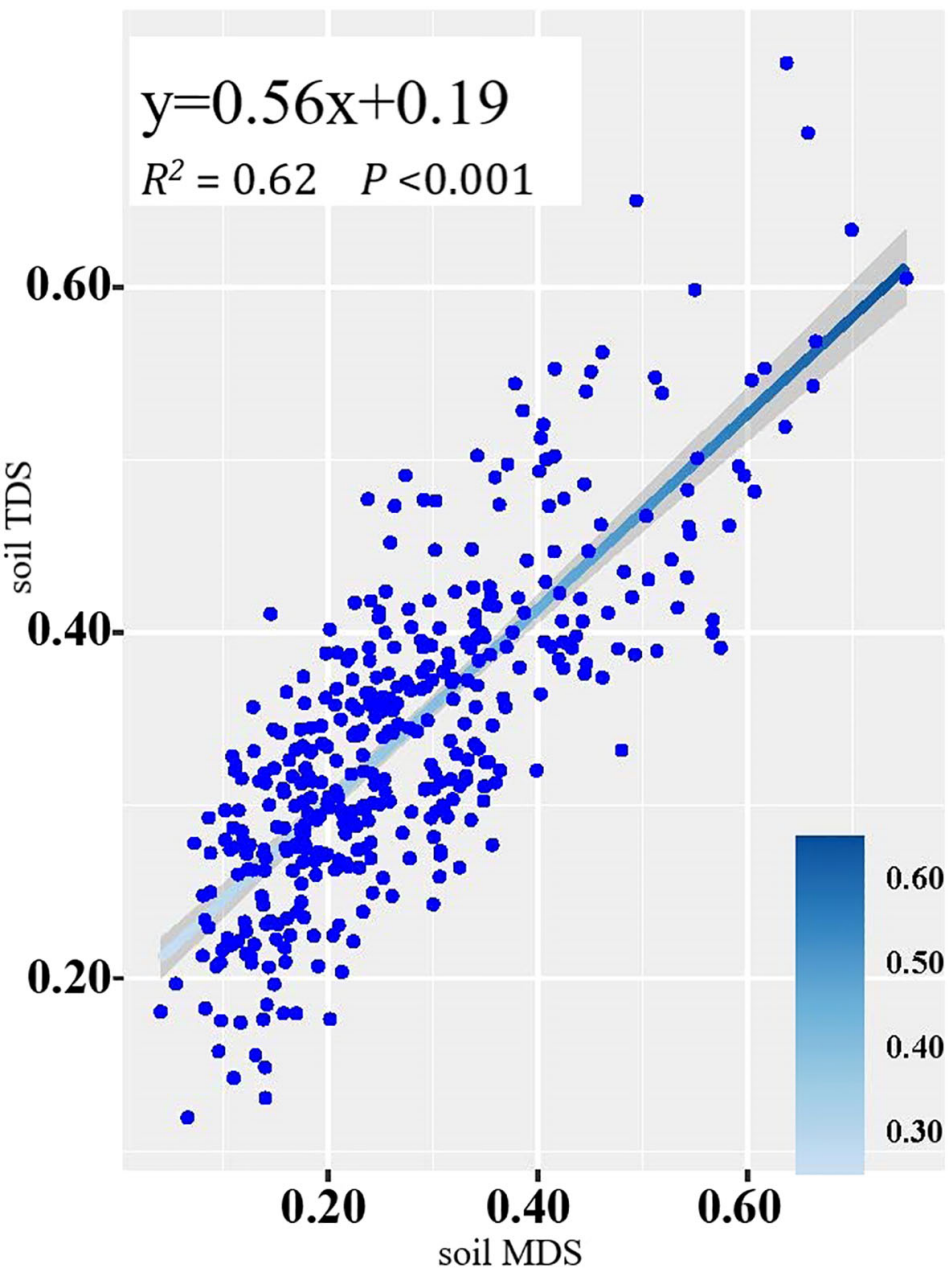
Linear fitting of *SPI*s based on the soil MDS and soil TDS.

The *SPI*s of the soil TDS and the soil MDS showed a significant positive correlation (*R^2^* = 0.62) (**Figure 3**), suggesting that the soil MDS could be substituted for the soil TDS.

### 3.2 Correlation between the soil MDS and species diversity

**Figure 4:**
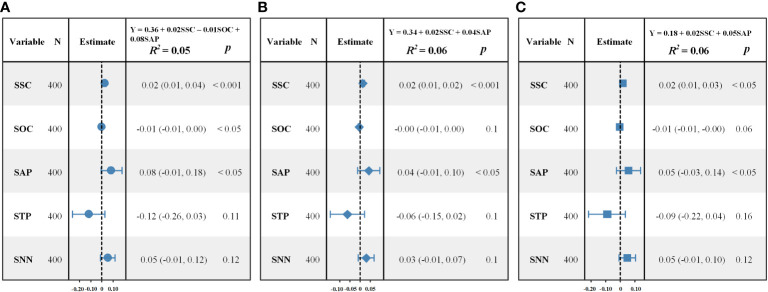
Forest plots of the observed and the predicted values of species diversity indices calculated by MLR. **(A)** Shannon–Wiener index; **(B)** Simpson index; **(C)** Pielou index.

**Figure 4** presented the results from the MLR models. SNN and STP had not entered into any of the models. The coefficients of determination (*R^2^*) were 0.05, 0.06 and 0.06 for the three MLR models, respectively, which indicated that the MLR models could explain less than 10% of the variance in species diversity.

**Figure 5:**
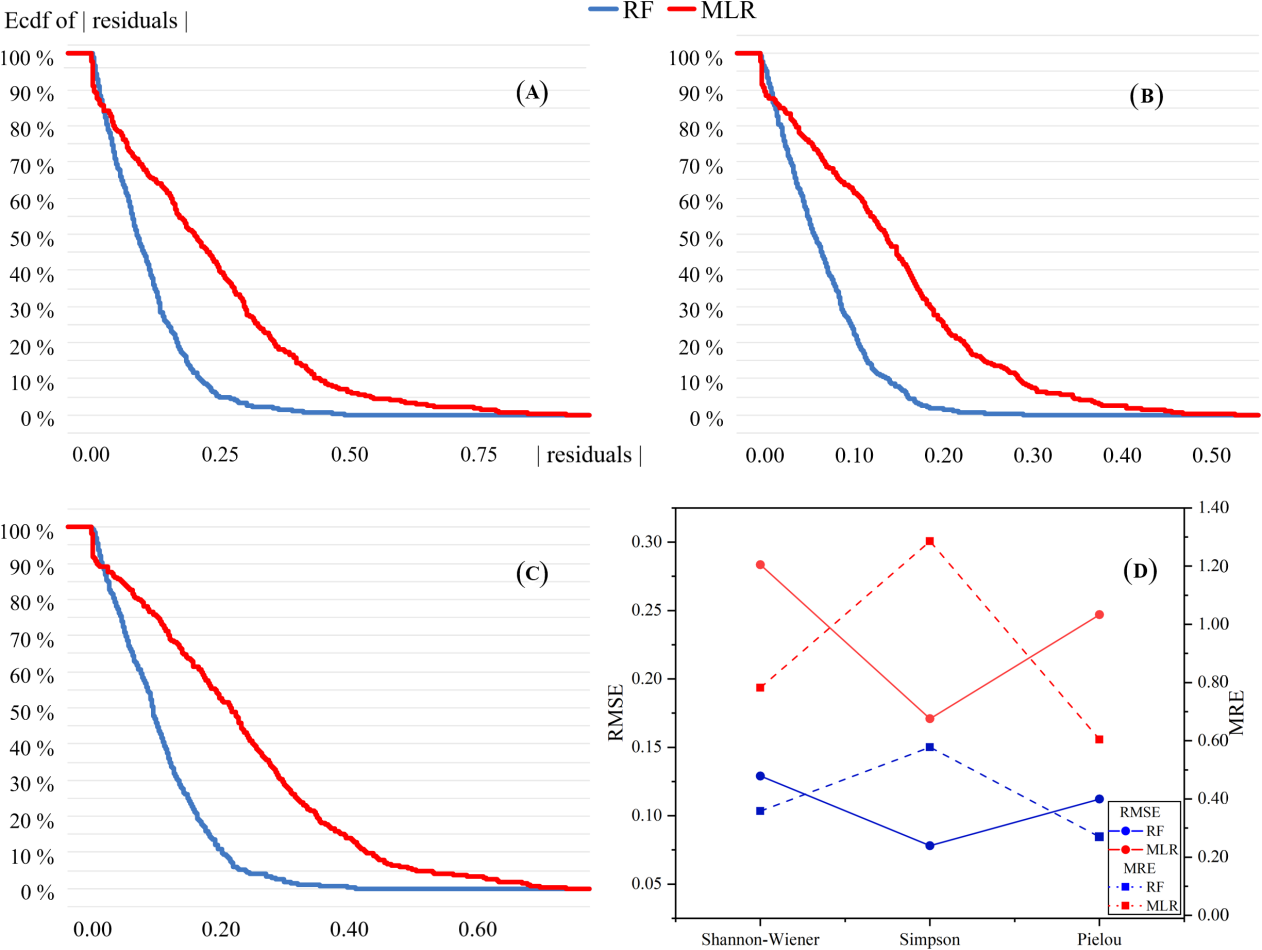
Reverse cumulative distribution of residuals of MLR and RF models. **(A–C)** represent Shannon–Wiener, Simpson and Pielou diversity indices, respectively. **(D)** is the results of RMSE and MRE of the two prediction models.

Mean relative errors (MREs) and the root mean square errors (RMSEs) were used to compare the accuracy of the MLR and RF models (**Figure 5**). The performance of the two models differed. The MLR models were better at predicting linear relationships while the RF models were more accurate in predicting non-linear relationships. The RF models performed significantly better than the MLR models in predicting the three species diversity indices. The values of RMSEs and MREs for all three indices in the RF models were substantially below than values in the MLR models. This suggested that the RF models predict more accurately than MLR models in species diversity according to the soil MDS. In addition, for each index, the residuals of the RF models were almost lower than those of MLR models, indicating that the RF models were significantly preferable to the MLR models in predicting species diversity distribution.

**Figure 6:**
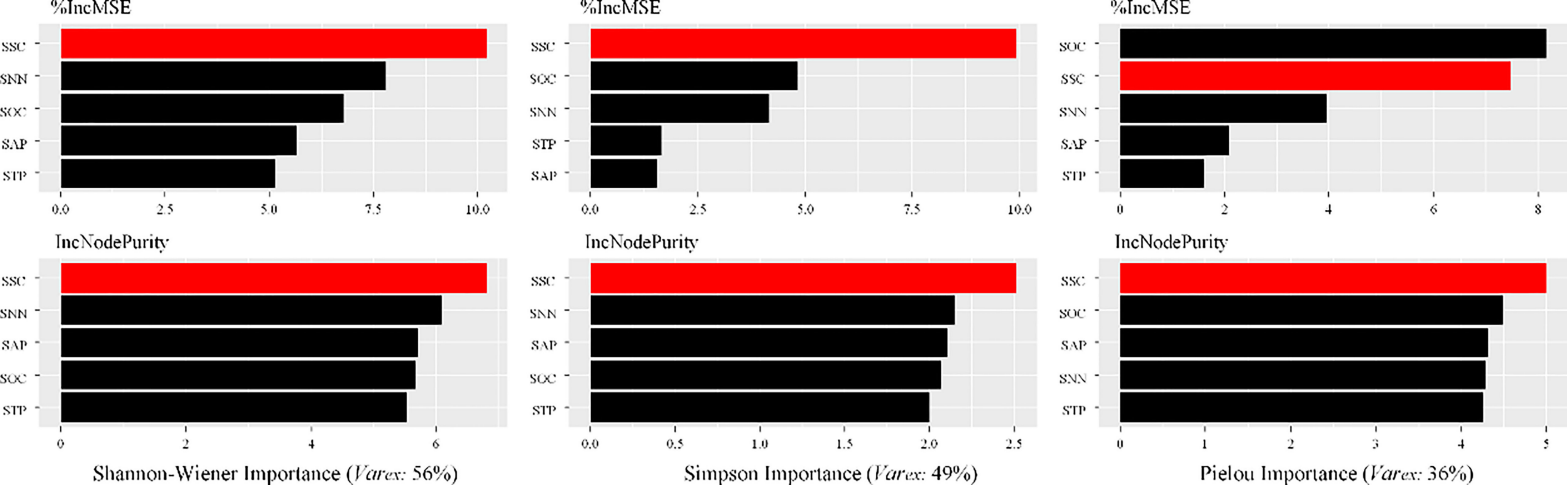
Contributions of the soil MDS indicator to the variability of species diversity measured using RF model.

The contributions of SOC, SSC, SAP, STP and SNN on the species diversity obtained from the RF models were shown in **Figure 6**. The explained variances of RF models were not same for different diversity indices. RF model had the highest explanation for the Shannon–Wiener index (56%), followed by the Simpson index (49%), and the Pielou index (36%). The relative contributions of the soil MDS indicators were different among three indices of species diversity. More specifically, the contribution of five MDS indicators to Shannon–Wiener index was ranked as follows: SSC > SNN > SOC (SAP) > SAP (SOC) > STP. For Simpson and Pielou indices, the contributions showed similar orders. Notably, SNN and SOC were important variables for explaining the variation in Simpson and Pielou indices, respectively. In total, SSC was the most influential soil MDS indicator in explaining the variability in species diversity, whereas STP was the least important factor (**Figure 6**).

### 3.3 Spatial variability and distribution

Parameters of original and predictive values of species diversity were given in **Table 4**. According to the *R^2^* and *RSS*, the exponential model has the best fitting to species diversity compared with linear, Gaussian and spherical models (**Supplementary Table S2**). After considering that RF model owned the higher explanation to the variance on the species diversity from soil MDS than MLR model, the RF-predictive diversity indices had been optimally fitted by using the exponential model.

**Table 4:** Parameters of semi-variograms predicted by RF models.

Indices	Variables	Model	C_0_(Nugget)	C_0_+C (Sill)	C_0_/(C_0_+C) (%)	Range (m)	*R^2^*	*RSS*
Shannon–Wiener	Original	Exp	0.43×10^-2^	0.08	5.38^***^	0.72	0.42	2.24×10^-4^
	RF-pre	Exp	0.28×10^-2^	0.03	9.33^***^	0.82	0.46	3.86×10^-5^
Simpson	Original	Exp	0.14×10^-2^	0.03	4.70^***^	0.77	0.37	4.20×10^-5^
	RF-pre	Exp	0.60×10^-3^	0.01	6.00^***^	0.90	0.42	7.23×10^-6^
Pielou	Original	Exp	0.25×10^-2^	0.06	4.17^***^	0.77	0.27	2.44×10^-4^
	RF-pre	Exp	0.13×10^-2^	0.02	6.50^***^	0.95	0.34	3.99×10^-5^

Exp is the exponential model. C_0_/(C_0_+C)< 25% (***) suggest a strong spatial dependence.

The semi-variograms of RF-predictive diversity indices were well structured with very small nugget effects, indicating that the sampling intervals were sufficient to measure the spatial variability. The nugget/sill ratios of the species diversity indices were all below 25% (4.17%-9.33%) with the autocorrelation ranged from 0.72 m (Shannon–Wiener index) to 0.95 m (RF-predictive Pielou index), indicating that species diversity showed high spatial dependence (**Table 4**). As the nugget/sill ratios were less than 75%, three species diversity indices can be further used to predict the values of un-sampled sites by interpolation method.

**Figure 7:**
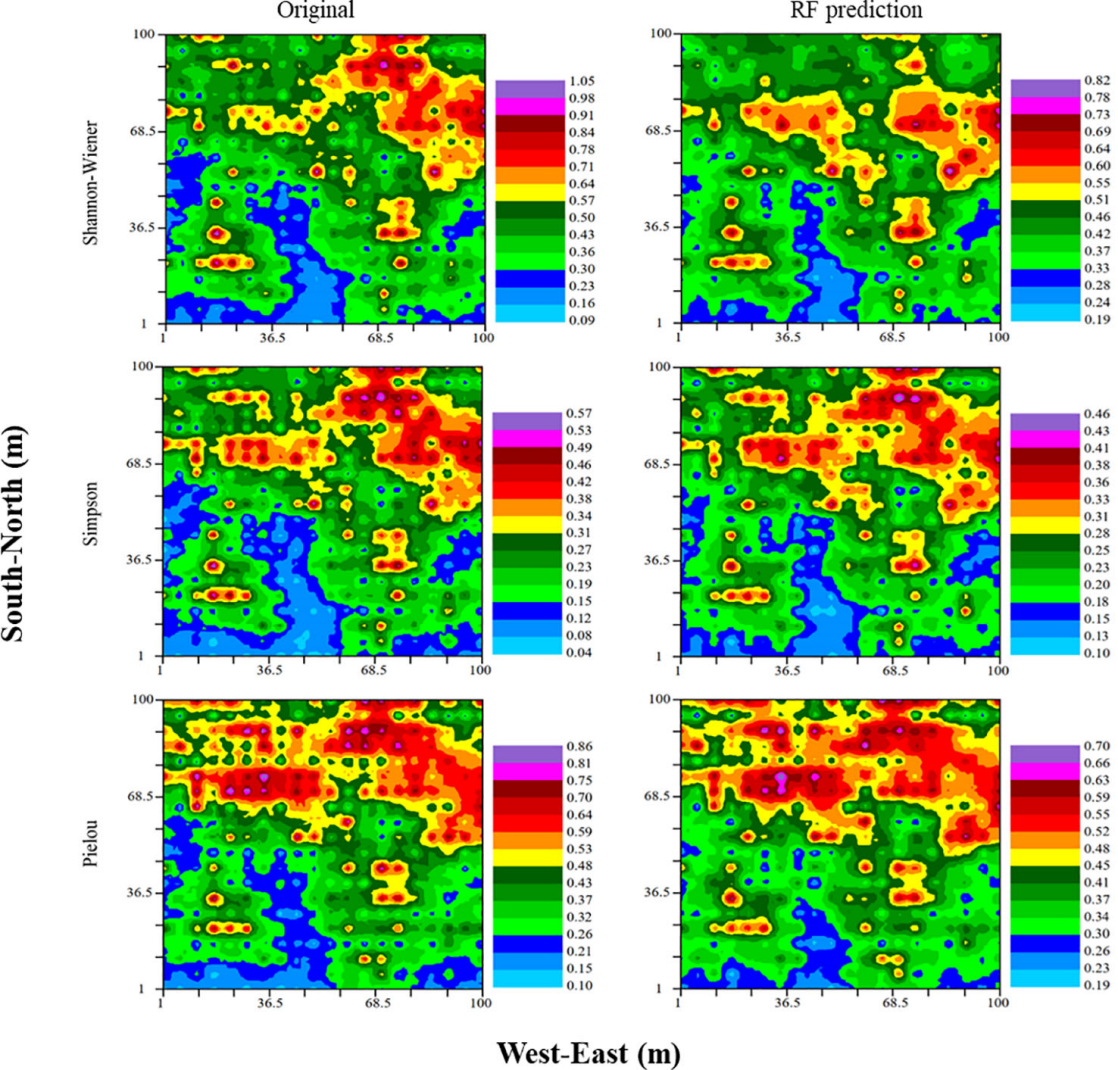
Spatial variations in species diversity interpolated using the original (left) and RF-predicted values (right), respectively.

Original and predictive kriging distribution maps were given in **Figure 7**. Spatial variations of Shannon–Wiener, Simpson and Pielou indices were largely consistent with their predicted values from RF models. In contrast, the prediction from MLR model was inappropriate, which undervalued the maximum and overvalued the minimum for three indices of species diversity (**Supplementary Figure S1**).

## 4 Discussion

### 4.1 The MDS composition of soil properties

The main limiting environmental factors in the arid desert ecosystem were water and salinity (Wang et al., 2020). Plant growth, nutrient cycling and the biological function were all affected by soil water content (SWC) and salinity (Smith and Doran, 1997; Yang et al., 2022). However, SWC was not included in the soil MDS. This is probably because the studied object (riparian forest) located close to the river, where SWC was relatively high compared to other desert regions, resulting in that the limiting effect of SWC on biological process was not obvious. Also, there was an obvious correlation between SWC and salinity in arid desert (Zhao et al., 2017). Soil salinity (SSC) entering the MDS meant that it removed SWC due to data redundancy. This can be proved in this paper that SSC showed a significant correlation with SWC.

Similar to the results of Gong et al. (2015) and Zhijun et al. (2018), STP was considered as the other limiting factor in the arid desert ecosystem, which included in the MDS because of its integral role in the biochemical reactions and nutrient cycling in plants (Marty et al., 2017; Shao et al., 2020). However, STP was not a valid indicator of the level of phosphorus availability. SAP was highly soluble, easily desorbed and rapidly exchanged. Therefore, SAP was one of the best properties of soil phosphorus supply, and was commonly used in the evaluation of soil fertility (Barbosa et al., 2014). SAP was also identified as the soil MDS indicators due to its higher Norm values within the group 3 (0.82). Nitrogen existed in soil *via* various forms such as ammonium and nitrate nitrogen, but the effects of different kinds of nitrogen on biological processes were different. In this study, SNN was included in the MDS, while neither STN nor SAN were included. This was because SNN can be directly and rapidly absorbed by plants compared with the other two types. SNN directly determined the ability of poor soil to supply nitrogen to plants in the short term (Zhou et al., 2020). SOC as a crucial element of soil (Ownley et al., 2003), contributed to the soil structural, biological and physical health, and was the basis of soil fertility (Yerima and Van Ranst, 2005). This was confirmed in our study that SOC was selected as a constituent of soil MDS in desert riparian forest.

Our results showed that the soil MDS consisted of SSC, SOC, STP, SAP and SNN. The correlation coefficient between *SPI*-MDS and *SPI*-TDS (*R* ^2^ = 0.62) indicated that our soil MDS can replace soil TDS in reflecting the soil property status. In other words, the constructed soil MDS could provide sufficient information to predict the spatial variation of plant diversity. The number of the soil properties was reduced to five by the soil MDS construction. Although some important soil information might be lost during the process, the soil MDS reduced the total number of indicators of the soil TDS, and provided a better example for other similar work.

### 4.2 Species diversity has high spatial dependence in desert riparian forests

Our results showed that the nugget/sill ratios (C_0_/(C_0_+C) of species diversity varied from 4.17% to 5.38%, indicating that species diversity has high spatial dependent in desert riparian forests. This might be caused by the fact that spatial variability of species diversity was largely dominated by structural factors rather than stochastic factors. This result was consistent with previous studies (Bhatti et al., 1991). The structure of diversity was mainly determined by soil conditions (Wild, 1971), topography (Bhatti et al., 1991) and climate (Berndtsson et al., 1993), because the continuous changes of these variables in space led to the regular structure of plant distribution and community composition. On the contrary, the stochastic factors that caused the spatial distribution of plants mainly referred to man-made or natural disturbances. Ebinur Lake Wetland was a well-known biological reserve in the western China. Governmental management made the local ecosystem rarely disturbed by human beings. At the same time, in a small range, such as 1 ha sampling plot in this study, the homogeneity of the environment made the natural disturbance will not easily appeared. Therefore, stochastic factors had not played a major role in the spatial variation of species diversity.

Our study also found that the range of species diversity varied from 0.72 m to 0.77 m (**Table 4**). This indicated that, species diversity owned a certain correlation between any two spatial locations within this range, but beyond this range, the interaction weakened with increasing distance (Zhao et al., 2015). The reason was that, plants have positive mutually beneficial or biased relations in a certain range, such as water redistribution and nutritional support from mothers to daughters, in order to increase the survive in the arid deserts (Schlesinger et al., 1996). The same result was also confirmed in the arid desert zone of Alashan Left Banner, China. Species diversity was similar in the range from 0.50 m to 0.82 m, but the association decreased continuously if beyond this distance (Ma et al., 2008).

### 4.3 Soil MDS affects spatial variability of species diversity

Our study suggested that soil salinity affected the spatial variability of species diversity. This was because soil salinity is one of the most important factors affecting plant distribution in arid desert regions (Chenchouni, 2017; Souahi et al., 2022). The scarce annual precipitation and the high osmotic pressure generated by perennial drought often led to increasing salt concentrations in the soil surface (Souahi et al., 2022), which in turn influenced the distribution of plants and the species composition of communities. It was well known that nutrient availability was one of the main determinants of plant diversity in arid desert regions (Reynolds and Haubensak, 2009). For example, Vourlitis et al. (2013) found that species diversity significantly increased with soil nutrients in Southern Mato Grosso, Brazil. A study from Zhang et al. (2010) in an arid desert demonstrated that 40% of the variation in plant diversity can be attributed to soil nutrients. This may be because plants prefer to grow in areas with higher nutrient availability in the arid desert. Such behavior would help them improve the chances of survival in the poor-soil environment (Al-Mutairi, 2022). This can be confirmed by the results of SOC in this study that SOC was the second factor determining the change of plant diversity. SOC was the most important indicator of soil nutrition, and its high value indicated that there were more nutrients available for plant to absorb and utilize in the soil (Souahi et al., 2022). In addition to SSC and SOC, SAP, STP and SNN were also affected spatial variation in species diversity in the arid desert regions. This may be because phosphorus was involved in various anti-stress activities of plants in arid desert, such as resistance to drought and salt stress. Compared with other nitrogen types, nitrate nitrogen can be directly absorbed by plants, so it can affect the spatial distribution of plants largely in poor-soil environments (Zhang, 2011). Similar results were found in Stohlgren et al. (2005) and Gu et al. (2003) that soil phosphorus and soil nitrogen had a greater impact on plant diversity than other factors in arid areas.

Our results confirmed that soil properties strongly affected the distribution of species diversity. The predicted species diversity based on the soil MDS using the RF model was basically consistent with the actual species diversity in desert riparian forests. Furthermore, the predictions of the highest and lowest values of species diversity were also similar to the actual values. This suggested that the soil MDS determined the spatial variation in species diversity. Our results also indicated that the RF models showed significantly greater comprehensiveness and accuracy in predicating the distribution of species diversity than MLR models. Earlier studies have indicated that RF model possesses more powerful modelling capabilities for complex interactions between indicators (Breiman, 2001; Guo et al., 2015; Xie et al., 2021). The relationship between soil properties and species diversity was very complex, because soil properties had not act on plant growth or distribution in one direction, but were intertwined with each other. Therefore, the accuracy of spatial distribution of species diversity predicted by RF model was bound to be higher than that by MLR (Zhang et al., 2017; Hamidi et al., 2021). In the further study, we suggested that nonlinear relationships rather than linear relationships should be considered more when exploring the influencing factors of species diversity.

Species diversity can be predicted using the soil MDS. This given us a good insight of how to dynamically monitor and assess changes in species diversity. Soil properties were relatively stable and measure easily, thus species diversity can be assessed by periodically examining soil properties. Similarly, since soil MDS significantly affected diversity, the ecological managers can increase species diversity by regulating constituents of the soil MDS (*i.e.*, SSC, STP, SAP, SOC and SNN), further improving ecosystem productivity and enhancing desert riparian ecosystem function.

## 5 Conclusions and perspectives

Our study identified that the soil MDS was consisted of SSC, STP, SOC, SAP and SNN in desert riparian forests of NW China. Based on the soil MDS, we compared traditional method (MLR) and machine learning algorithms (RF) model for predicting species diversity in typical desert riparian forest. The accuracy of the RF model was far superior to the MLR model. Our study found that RF predictions based on the soil MDS were visually highly matched to the original distribution of species diversity. Predicting plant diversity through the soil MDS was highly feasible. The MDS of soil properties determined the spatial variation in plant species diversity. SSC and SOC contributed decisively to the distribution of species diversity. STP also had minor effect on the spatial distribution of species diversity. These findings can provide essential references for biodiversity conservation and sustainable development in the ELWNR and other low productivity drylands around the world.

Productivity, as one of the important ecosystem functions, is closely related to community species composition and diversity. The relationship between species diversity and productivity in desert riparian forests is also a question that deserves to be investigated. However, we did not test it in this study. In the future, we can explore the plant diversity and biomass dynamics and their relationship with the environment in arid desert areas combining abiotic factors such as precipitation, temperature, topography and soil properties.

## Data availability statement

The data analyzed in this study is subject to the following licenses/restrictions: The datasets used or analysed during the current study are available from the corresponding author on reasonable request. Requests to access these datasets should be directed to lixiaotong@stu.xju.edu.cn.

## Author contributions

Conceptualization, YC and XL; Methodology, YC and XL; Software, YC and XL; Validation, JW, YC, HW, LJ, and XL; Formal Analysis, YC and XL; Investigation, YC, JW, LJ, and HW; Resources, YC, GL, XY, and LJ; Data Curation, YC and XL; Writing – Original Draft Preparation, XL; Writing – Review & Editing, YC, JW, LJ, HW, XY and XL; Visualization, YC and XL; Supervision, GL and XY; Project Administration, GL, JW and LJ. All authors contributed to the article and approved the submitted version.

## Funding

This study was financially supported by National Natural Science Foundation of China (No.42171026) and Xinjiang Uygur Autonomous Region innovation environment Construction special project & Science and technology innovation base construction project (PT2107).

## Acknowledgments

We greatly thank Hanpeng Li, Wenjing Li, Zhoukang Li, Kunduz Sattar and Shiyun Wang et al. for their strong help with field and laboratory work.

## Conflict of interest

The authors declare that the research was conducted in the absence of any commercial or financial relationships that could be construed as a potential conflict of interest.

## Publisher’s note

All claims expressed in this article are solely those of the authors and do not necessarily represent those of their affiliated organizations, or those of the publisher, the editors and the reviewers. Any product that may be evaluated in this article, or claim that may be made by its manufacturer, is not guaranteed or endorsed by the publisher.
