# Supplementary material for: Predicting spatial variability of species diversity with the minimum data set of soil properties in an arid desert riparian forest
Source: Front Plant Sci. 2022 Nov 11;13:1014643. doi: 10.3389/fpls.2022.1014643 (PMC9691764; doi:10.3389/fpls.2022.1014643)
Supplement: Supplementary file 1 [file DataSheet_1.docx]

**Predicting spatial variability of species diversity with the minimum data set of soil properties in an arid desert riparian forest**

Xiaotong Li^1,2,3†^, Yudong Chen^1,2,3†^, Guanghui Lv^1,2,3*^, Jinlong Wang^1,2,3^, Lamei Jiang^1,2,3^, Hengfang Wang^1,2,3^, Xiaodong Yang^4*^

1 College of Ecology and Environment, Xinjiang University, Xinjiang, China;

2 Key Laboratory of Oasis Ecology of Education Ministry, Xinjiang University, Xinjiang, China;

3 Xinjiang Jinghe Observation and Research Station of Temperate Desert Ecosystem, Ministry of Education, Jinghe, China.

4 School of Civil & Environmental Engineering and Geography Science, Ningbo University, Ningbo, China.

^†^ Those authors contributed equally.

*Correspondence: Guanghui Lv, guanghui_xju@sina.com; and Xiaodong Yang, xjyangxd@sina.com

**Table S1** Frequency of plant species in study area

| Species | Frequency | Species | Frequency |
| --- | --- | --- | --- |
| *Phragmites australis* | 91.71% | *Suaeda microphylla* | 0.19% |
| *Apocynum venetum* | 2.90% | *Alhagi sparsifolia* | 0.12% |
| *Halimodendron halodendron* | 1.76% | *Reaumuria soongorica* | 0.11% |
| *Nitraria tangutorum* | 1.71% | *Haloxylon ammodendron* | 0.04% |
| *Achnatherum splendens* | 0.72% | *Salsola collina* | 0.04% |
| *Lycium ruthenicum* | 0.42% | *Sonchus oleraceus* | 0.03% |
| *Populus euphratica* | 0.23% | *Glycyrrhiza uralensis* | 0.02% |

**TABLE S2** Parameters of semi-variograms tested using the RF models.

| Indices | Variables | Model | C_0_  (Nugget) | C_0_+C (Sill) | C_0_/(C_0_+C) (%) | Range (m) | *R^2^* | *RSS* |
| --- | --- | --- | --- | --- | --- | --- | --- | --- |
| Shannon–Wiener | Original | Exp | 0.43×10^-2^ | 0.08 | 5.38^***^ | 0.72 | 0.42 | 2.24×10^-4^ |
|  |  | Lin | 7.00×10^-2^ | 0.09 | 77.78* | 12.31 | 0.38 | 8.36×10^-5^ |
|  |  | Sph | 0 | 0.08 | 0^***^ | 1.80 | 0.33 | 2.85×10^-4^ |
|  |  | Gau | 0.50×10^-2^ | 0.08 | 6.25^***^ | 0.88 | 0.33 | 2.57×10^-1^ |
|  | RF-pre | Exp | 0.28×10^-2^ | 0.03 | 9.33^***^ | 0.82 | 0.46 | 3.86×10^-5^ |
|  |  | Lin | 2.60×10^-2^ | 0.03 | 8.67^***^ | 12.31 | 0.38 | 8.83×10^-6^ |
|  |  | Sph | 0 | 0.03 | 0^***^ | 1.86 | 0.33 | 4.69×10^-5^ |
|  |  | Gau | 0.20×10^-2^ | 0.03 | 6.67^***^ | 0.92 | 0.34 | 4.66×10^-5^ |
| Simpson | Original | Exp | 0.14×10^-2^ | 0.03 | 4.70^***^ | 0.77 | 0.37 | 4.20×10^-5^ |
|  |  | Lin | 2.40×10^-2^ | 0.03 | 8^***^ | 12.31 | 0.31 | 1.26×10^-5^ |
|  |  | Sph | 0 | 0.03 | 0^***^ | 1.82 | 0.27 | 4.78×10^-5^ |
|  |  | Gau | 0.10×10^-2^ | 0.03 | 3.33 | 0.89 | 0.27 | 4.77×10^-5^ |
|  | RF-pre | Exp | 0.60×10^-3^ | 0.01 | 6.00^***^ | 0.90 | 0.42 | 7.23×10^-6^ |
|  |  | Lin | 0.90×10^-2^ | 0.01 | 90^*^ | 12.31 | 0.29 | 1.31×10^-6^ |
|  |  | Sph | 0 | 0.01 | 0^***^ | 1.90 | 0.28 | 8.71×10^-6^ |
|  |  | Gau | 0 | 0.01 | 0^***^ | 0.93 | 0.28 | 8.67×10^-6^ |
| Pielou | Original | Exp | 0.25×10^-2^ | 0.06 | 4.17^***^ | 0.77 | 0.27 | 2.44×10^-4^ |
|  |  | Lin | 4.90×10^-2^ | 0.07 | 7^***^ | 12.31 | 0.16 | 4.67×10^-5^ |
|  |  | Sph | 0 | 0.06 | 0^***^ | 1.77 | 0.18 | 2.72×10^-4^ |
|  |  | Gau | 0.20×10^-2^ | 0.06 | 3.33 | 0.86 | 0.18 | 2.72×10^-4^ |
|  | RF-pre | Exp | 0.13×10^-2^ | 0.02 | 6.50^***^ | 0.95 | 0.34 | 3.99×10^-5^ |
|  |  | Lin | 1.70×10^-2^ | 0.02 | 85.00* | 0.30 | 0.31 | 5.10×10^-6^ |
|  |  | Sph | 0 | 0.02 | 0^***^ | 1.00 | 0.18 | 4.72×10^-5^ |
|  |  | Gau | 0 | 0.02 | 0^***^ | 0.99 | 0.19 | 4.71×10^-6^ |

Exp, exponential model; Lin, linear model; Sph, spherical model; Gau, gaussian model. C_0_/(C_0_+C) < 25% (***), 25% < C_0_/(C_0_+C) < 75% (**), and C_0_/(C_0_+C) > 75% (*) suggest a strong, moderate, and weak spatial dependence, respectively.


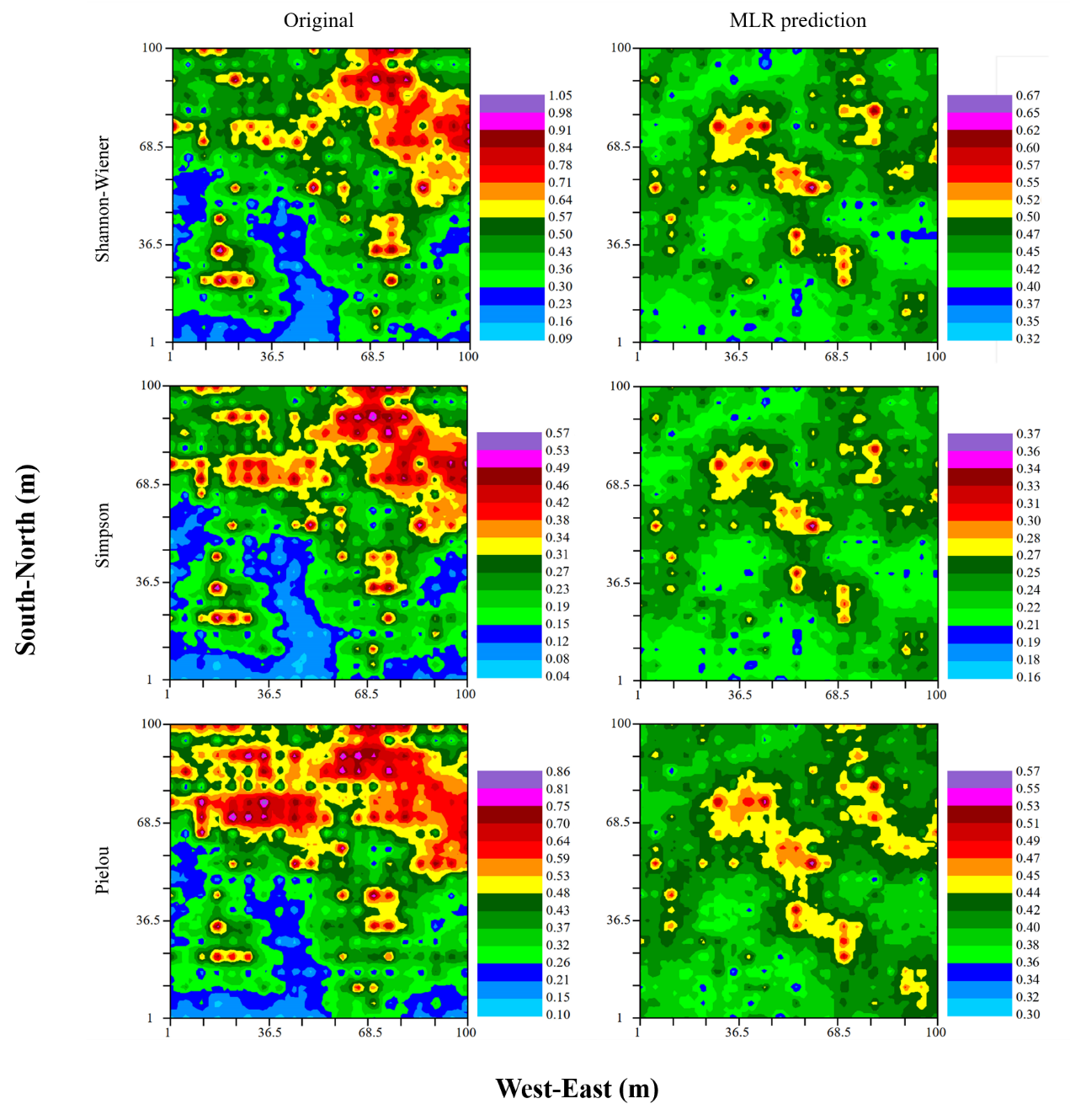


**Figure S1** Spatial variations in species diversity interpolated using the original (left) and MLR-predicted values (right), respectively.
